# CKD and Hospitalization in the Elderly: A Community-Based Cohort Study in the United Kingdom

**DOI:** 10.1053/j.ajkd.2010.09.026

**Published:** 2011-05

**Authors:** Dorothea Nitsch, Bareng A.S. Nonyane, Liam Smeeth, Christopher J. Bulpitt, Paul J. Roderick, Astrid Fletcher

**Affiliations:** 1 Faculty of Epidemiology and Population Health, London School of Hygiene and Tropical Medicine, Bloomsbury, London, UK; 2 Care of the Elderly, Imperial College Hammersmith Campus, Hammersmith, London, UK; 3 Public Health Sciences and Medical Statistics, University of Southampton, Southampton General Hospital, Southampton, UK

**Keywords:** Chronic kidney disease, cohort study, dipstick proteinuria testing, general population, hospitalization, older people

## Abstract

**Background:**

We previously have shown that chronic kidney disease (CKD) is associated with cardiovascular and all-cause mortality in community-dwelling people 75 years and older. The present study addresses the hypothesis that CKD is associated with a higher rate of hospital admission at an older age.

**Study Design:**

Cohort study.

**Setting & Participants:**

15,336 participants from 53 UK general practices underwent comprehensive health assessment between 1994 and 1999.

**Predictor:**

Data for estimated glomerular filtration rate (eGFR, derived from creatinine levels using the CKD Epidemiology Collaboration [CKD-EPI] study equation) and dipstick proteinuria were available for 12,371 participants.

**Outcomes:**

Hospital admissions collected from hospital discharge letters for 2 years after assessment.

**Measurements:**

Age, sex, cardiovascular risk factors, possible biochemical and health consequences of kidney disease (hemoglobin, phosphate, and albumin levels; physical and mental health problems).

**Results:**

2,310 (17%) participants had 1 hospital admission, and 981 (7%) had 2 or more. After adjusting for age, sex, and cardiovascular risk factors, HRs were 1.66 (95% CI, 1.21-2.27), 1.17 (95% CI, 0.95-1.43), 1.08 (95% CI, 0.90-1.30), and 1.11 (95% CI, 0.91-1.35) for eGFRs <30, 30-44, 45-59, and ≥75 mL/min/1.73 m^2^, respectively, compared with eGFRs of 60-74 mL/min/1.73 m^2^ for hospitalizations during <6 months of follow-up. HRs were weaker for follow-up of 6-18 months. Dipstick-positive proteinuria was associated with an increased HR throughout follow-up (HR, 1.29 [95% CI, 1.11-1.49], adjusting for cardiovascular risk factors). Dipstick-positive proteinuria and eGFR <30 mL/min/1.73 m^2^ were independently associated with 2 or more hospital admissions during the 2-year follow-up. Adjustment for other health factors and laboratory measurements attenuated the effect of eGFR, but not the effect of proteinuria.

**Limitations:**

Follow-up limited to 2 years, selection bias due to nonparticipation in study, missing data for potential covariates, and single noncalibrated measurements from multiple laboratories.

**Conclusions:**

The study indicates that community-dwelling older people who have dipstick-positive proteinuria and/or eGFR <30 mL/min/1.73 m^2^ are at increased risk of hospitalization.

Chronic kidney disease (CKD) is a major health problem and is associated with cardiovascular and all-cause mortality.^1-3^ There are few data about the overall effects of CKD on morbidity and overall health outcomes in the general population. A large study in the United States has shown that for those who are insured, individuals with CKD appear to be at higher risk of later hospitalization.^2^ However, such a study has a number of limitations. In particular, assessment of kidney function was not available for the entire population, but was measured selectively in people who had a clinical indication. This means that people included in the study potentially were unrepresentative of the population, making results difficult to generalize. In addition, such database studies have limited data for confounding and explanatory factors.

In the United Kingdom, there are limited data for hospital admissions for CKD. Using a large study of older people living in the community, we previously have shown that CKD is associated with a high burden of comorbid conditions^4^ and higher risk of death that was independent of concurrent comorbid conditions and conventional cardiovascular risk factors.^1^ In the present article, we report results for the association of CKD with rate of hospital admission.

## Methods

### Study Design

We used data from a cluster randomized trial of older people in the setting of general practice (the Medical Research Council [MRC] Study of Multidimensional Assessment of Older People). The study protocol and main results have been reported previously.^5,6^ In brief, this trial compared 2 methods of multidimensional assessment (universal vs targeted assessment) in people 75 years and older registered in 106 general practices from the MRC General Practice Research Framework in England, Wales, and Scotland selected to be representative of the UK general practice standardized mortality ratios and Jarman deprivation score (categorized into low, middle, and high scores).^7^ All patients 75 years or older registered with the practices were eligible and invited to participate unless they were resident in long-stay hospitals or nursing homes or had a terminal illness. The trial was approved by relevant local ethics committees. This report uses data from the 53 practices in the universal arm of the trial because in this arm, all patients were offered an in-depth health assessment, including a routine blood test. In the universal arm, 15,336 of 20,934 (73.2%) participants attended for the assessment; nonresponders were older and more likely to be women.^5^

### Data Collected at Assessment

Assessments were conducted in 1994-1999 and carried out by nurses trained in the study methods and assessments following a structured questionnaire and protocol.^5^

Patients' height, weight, waist and hip circumferences, and blood pressure (average of 2 measurements each) were measured. A nonfasting blood sample was obtained for a biochemical screen that included serum creatinine, urea, potassium, albumin, calcium, phosphate, bilirubin, alkaline phosphatase, aspartate aminotransferase, and urate and a full blood cell count (hemoglobin, white blood cell count, and platelets). Urine dipstick for protein, glucose, and blood was performed, and if positive for protein, a midstream urine sample was obtained. Sociodemographic information, self-reported medical history, lifestyle, and medication data were obtained using nurse interview. Medication data were derived from participants bringing their medicines to the assessment, and drugs were coded into broad classes using the British National Formulary chapter headings. Diabetes was classified according to self-report of a medical diagnosis, use of antidiabetic medication, or the presence of a high random blood glucose level. Participants were asked about alcohol consumption, smoking history, and perception of physical activity. Activities of daily living (ADLs) were categorized by the number of dependencies for 8 activities (washing, dressing, cutting toe nails, cooking, shopping, doing light housework, walking 50 yards, and going up and down stairs and steps). We defined full or partial dependency as being unable to perform 2 or more ADLs. A score <24 on the Mini-Mental State Examination^8^ was considered to indicate cognitive impairment, and a score >5 on the Geriatric Depression Scale,^9^ significant depression. Other variables included self-reported history of cancer, unexpected weight loss of more than half a stone (1/2 stone = 3.175 kg), and history of falls in last 6 months (<2 vs ≥2).

### Kidney Function

Of 45 local laboratories to which serum samples were sent, 37 used the modified Jaffé method and 7 used an enzymatic method for serum creatinine (in 1, the method was unknown).

The CKD Epidemiology Collaboration (CKD-EPI) Study equation^10,11^ was used to calculate estimated glomerular filtration rate (eGFR) and categorized into eGFR groups^12^ of ≥75, 60-74, 45-59, 30-44, <30 mL/min/1.73 m^2^ using nonstandardized serum creatinine multiplied by 0.95 (this represents the difference between standardized and nonstandardized creatinine in the Modification of Diet in Renal Disease [MDRD] Study laboratory).^13^ Only those who had dipstick data and no evidence of urinary tract infection on the midstream urine sample were defined as having available urine dipstick data. Dipstick proteinuria was categorized as none/trace versus proteinuria (+, ++, and +++).

### Outcomes

Information for hospital admissions (defined as a stay of at least 1 night) for a 2-year period after the date of assessment was collected from the hospital discharge letter extracted from the practice records. Diagnostic codes for hospital admission were summarized using *International Classification of Diseases, Tenth Revision* chapter headings as circulatory (I00-I99), cancer or diseases of the blood system (C00-D89), of infectious origin (A00-B99, L00-L08, K65, M00-M03, and J00-J22), or other causes of hospital admissions. Multiple causes were allowed. Study participants were registered with the Office for National Statistics for mortality follow-up (date and cause of death).

### Data Analysis

Data analyses were performed using Stata, version 11 (www.stata.com). Crude associations for eGFR and dipstick proteinuria with baseline criteria (using χ^2^ tests and tests for trend as appropriate) and for hospitalization rates (with corresponding 95% confidence intervals [CIs]) were calculated. We censored participants at death (if it occurred outside the hospital) or the end of the 2-year follow-up after the health assessment.

A Cox proportional hazards model was used to model the outcome of time to first hospitalization after the baseline assessment. The proportional hazards assumption of the Cox hazard model did not hold for the entire follow-up of 2 years because there was a time-varying effect of eGFR, age, and sex, which changed rapidly within the first few months. In other words, there were strong selection effects over time that led to a changing hazard ratio (HR) dependent on the time of follow-up. Follow-up time therefore was divided into 2 periods (or time bands) for each patient: the first 6 months after the baseline assessment and the period from 6 months to 2 years (the end of follow-up). Cox proportional hazard models were fitted separately for the time from the start of the study up to 6 months and separately from 6 months to 24 months for those who were not hospitalized within 6 months and were alive (with baseline factors and measurements carried forward to the 6-month start).

All models were adjusted for the effect of age using 4 strata with cutoff points at ages 80, 85, and 90 years (model 1 in tables). We tested for interactions between eGFR and dipstick proteinuria and for eGFR and sex by fitting the respective interaction terms in the age-adjusted models and performing Wald test for exclusion of all interaction terms (in both the 6-month and 6- to-24 month follow-up cohorts). Model 2 adjusted for socioeconomic status, comorbid conditions, cardiovascular risk, or use of cardiovascular drugs. Variables were Jarman score, smoking status (current, ex-, or never smoker), alcohol use (never , ex-, and current drinker), self-reported physical activity (very active, fairly, not very, and not at all), waist-to-hip ratio (sex-specific quintiles), and comorbid conditions using self-reported history of cardiovascular disease (CVD; heart attack or stroke) and diabetes. Hypertension was modeled separately as physician-diagnosed hypertension, average blood pressure >140/90 mm Hg, or antihypertensive drug use. Cardiovascular drugs were statins or aspirin. We adjusted for angiotensin-converting enzyme–inhibitor and angiotensin II receptor blocker use separately from other antihypertensive agents. We ran models 1 and 2 separately for both eGFR and dipstick proteinuria and further ran models with both eGFR and proteinuria included.

We then added laboratory measurements (model 3) and other health measures (model 4) to investigate their role in the observed associations. Hemoglobin and phosphate levels were categorized as quintiles for each sex, and albumin, as quintiles for each sex and assay type. Other health measures were Mini-Mental State Examination scores (≤23 vs >23), Geriatric Depression Scale score (≤5 vs >5), overall health perception (poor vs not poor), and ADLs.

For those hospitalized, we derived separate dummy variables for the presence of infectious, cancer, cardiocirculatory, or other causes for hospitalization. Logistic regression analyses were carried out for the odds for specific causes of hospitalization (relative to not having this cause) as a function of measurements preceding that hospitalization (adjusted for age and sex). Secondary analysis was conducted for the association of CKD with total number of admissions (categorized as 0, 1, or ≥2) during follow-up using a multinomial logistic regression. In all models, robust standard errors were calculated to account for the study design of 53 practices from which participants were recruited.

## Results

### Crude Associations With Subsequent Hospitalization

For 13,177 of 15,336 (86%) participants who completed the in-depth assessment, eGFR was calculated. Missing data included patient refusal of phlebotomy, poor veins, lost blood sample, or unknown. During the 2-year follow-up, 3,291 of 13,177 (25%) participants with eGFR data had at least 1 hospital admission; 2,310 (17%) had only 1 admission and 981 (7%) had 2 or more admissions. For those hospitalized at least once, the next admission occurred within a median of 98 (25th-75th percentile, 42-225) days. There were 12,371 participants who had both eGFR and dipstick proteinuria data. Six participants died on the day of admission to the hospital, and 2,279 died after being admitted to the hospital. There were 999 patients who died within 2 years of follow-up without entering the hospital; these were censored for the analysis at their death date.

Selected baseline characteristics and their associations with eGFR and proteinuria are listed in Table 1. Associations of baseline characteristics with subsequent hospital admissions are listed in Table 2. Hospitalization rates increased with increasing age, and men were more likely to be hospitalized than women. When investigating causes of admissions, 23.2% of all admissions were for circulatory reasons, 14.2% had infections as a contributing cause, and 11.6% had cancer or blood-related diseases as a contributing cause.

### Associations of eGFR and Proteinuria With Subsequent Hospitalization

Both decreasing categories of eGFR and dipstick-positive proteinuria had higher crude hospitalization rates (Table 2). Hospitalization rate ratios (age adjusted) stratified by sex and dipstick proteinuria results are listed in Table 3. For those hospitalized, there was no evidence for a trend across eGFR categories to have more infectious disease (*P* = 0.8) or cancer diagnoses (*P* = 0.5; adjusted for age and sex). However, for those hospitalized, in age- and sex-adjusted analyses, there was a trend (*P* < 0.001) for those with lower eGFRs to have more hospitalizations related to CVD, with odds ratios (ORs) of 1.86 (95% CI, 1.19-2.92), 1.58 (95% CI, 1.25-1.99), 1.29 (95% CI, 1.03-1.62), and 0.91 (95% CI, 0.72-1.15) for eGFRs <30, 30-44, 45-59, and ≥75 mL/min/1.73 m^2^, respectively, compared with eGFRs of 60-74 mL/min/1.73 m^2^. These associations were not confounded by dipstick proteinuria, which was not associated with infections, cancer, or circulatory reasons (data not shown).

Subsequent analyses listed in Table 4 were based on people with complete information for confounding variables (n = 10,977); results for analyses with all data with varying totals for each model are very similar (data not shown). Adjusting for age and sex of participants, we found a strong effect of eGFR <30 mL/min/1.73 m^2^, which was stronger in the first 6 months of follow-up compared with the subsequent 18 months (model 1). In age-adjusted analysis, there was no evidence for an interaction of eGFR and sex in up to 6 months' follow-up (*P* = 0.7) and during the subsequent 18 months of follow-up (*P* = 0.8). The association of eGFR <30 mL/min/1.73 m^2^ with hospitalization attenuated, but remained significant, when adjusting further for all cardiovascular risk factors and underlying CVD, as well as Jarman score (model 2). HRs for eGFR <30 mL/min/1.73 m^2^ during the first 6-month period were confounded weakly by dipstick positivity. There was no evidence for an interaction of eGFR and dipstick positivity in up to 6 months of follow-up (*P* = 0.7) and during the subsequent 18 months of follow-up (*P* = 0.6). There was no evidence of time-dependent effects of dipstick positivity, and the age- and CVD risk–adjusted HR was 1.29 (95% CI, 1.11-1.49) for the total 2-year follow-up.

We then adjusted the model with both eGFR and proteinuria for hemoglobin, albumin, and phosphate levels (model 3), and the association of eGFR <30 mL/min/1.73 m^2^ was attenuated further by 12% (for eGFR <30 mL/min/1.73 m^2^; HR, 1.44 [95% CI, 1.04-1.98] in the first 6 months and 1.06 [95% CI, 0.81-1.40] in the subsequent 18 months; the reference group is eGFR of 60-74 mL/min/1.73 m^2^; other data not shown). Adjustment for hemoglobin, albumin, and phosphate levels did not appreciably alter the effects of dipstick proteinuria in the same models (HR, 1.23 [95% CI, 0.97-1.57] in the first 6 months and HR, 1.28 [95% CI, 1.06-1.53] in the subsequent 18 months).

Further adjustments for Mini-Mental State Examination scores, Geriatric Depression Scale scores, overall health perception, and ADLs attenuated the association of eGFR <30 mL/min/1.73 m^2^ with hospitalization (HR, 1.28 [95% CI, 0.91-1.80] for the first 6 months and HR, 1.01 [95% CI, 0.76-1.34] for the subsequent 18 months), whereas in the same analysis, the association of proteinuria with hospitalization remained very similar (HR, 1.21 [95% CI, 0.95-1.55] in the first 6 months and HR, 1.27 [95% CI, 1.05-1.53] for the subsequent 18 months; model 4; other data not shown).

### Associations of eGFR and Proteinuria With Number of Hospitalizations

Compared with eGFR of 60-74 mL/min/1.73 m^2^, eGFR categories of 30-44 and <30 mL/min/1.73 m^2^ were associated with increased ORs for 2 or more hospitalizations during the 2-year follow-up. For those with eGFR <30 mL/min/1.73 m^2^ in particular, there was a more than doubled OR (Table 5). Adjustments for cardiovascular risk factors at baseline attenuated associations of eGFR with number of hospitalizations, with an increased OR remaining for only eGFR <30 mL/min/1.73 m^2^ and 2 or more admissions. Dipstick-positive proteinuria (not adjusted for eGFR) was associated with multiple hospitalizations during follow-up; adding potential cardiovascular confounding variables did not attenuate the association appreciably. In a model with both proteinuria and eGFR, we found that both dipstick-positive proteinuria and eGFR <30 mL/min/1.73 m^2^ were associated independently with the odds of multiple hospitalizations during the 2-year follow-up, even after adjustment for CVD (Table 5).

Laboratory parameters (hemoglobin, phosphate, and albumin) attenuated the observed associations for eGFR <30 mL/min/1.73 m^2^ (OR, 1.21 [95% CI, 0.79-1.85] for 2 or more hospitalizations compared with none), whereas the effect for dipstick proteinuria was completely unchanged (model 3). Additional adjustments for other health factors (model 4) attenuated the association of number of hospitalizations with eGFR <30 mL/min/1.73 m^2^ (OR, 1.16 [95% CI, 0.73-1.85] for 2 or more hospitalizations compared with none), whereas the association with dipstick proteinuria was unchanged (OR, 1.34 [95% CI, 1.04-1.71] for 2 or more hospitalizations compared with none).

## Discussion

Our results show that dipstick-positive proteinuria is associated with an approximate 30% increased risk of single and multiple hospitalizations during the 2 years after measurement. We found a strong association of eGFR <30 mL/min/1.73 m^2^ with the short-term incidence of hospitalization (<6 months) and a 50% increase in odds of more than 1 admission. This finding agrees with previous studies that examined only eGFR.^2,14^ Our results indicate the potential importance of dipstick testing and eGFR measurement in the early identification of older people who are at risk of hospitalization.

Other studies in the United States using health insurance claims data have found broadly similar results of an association of eGFR with risk of subsequent hospitalization.^2,15^ However, many people with less severe degrees of CKD are not identifiable in US claims databases, limiting their utility.^16^ In the United Kingdom, to our knowledge, no community-based study has investigated whether CKD increases the risk of hospitalization.

It is unclear exactly what explains the associations found. Rate ratios for eGFR across the total follow-up were less marked for men than women (Table 3); however, these rate ratios conceal variations in the pattern of HRs during follow-up. Men, those who were older, and those with lower eGFRs had higher admission rates during the first few months of follow-up. For this reason, we split the time into a short- (up to 6 months) and long-term risk period (6-18 months). Adjustment for concurrent CVD risk factors attenuated the associations of eGFR <30 mL/min/1.73 m^2^ for the first 6 months of follow-up, suggesting that cardiovascular risk factors may explain some (but not all) associations. Adjustment for hemoglobin, phosphate, or albumin levels led to little attenuation of the association of eGFR with time to hospitalization; in contrast, there was more attenuation of the association of eGFR with number of hospitalizations. Further adjusting for health factors that indicate other aspects of physical and mental health almost completely attenuated the association of eGFR <30 mL/min/1.73 m^2^ with either rate or number of admissions. It therefore is possible that some of the association of eGFR <30 mL/min/1.73 m^2^ may be mediated through factors related to other health problems. No such attenuation was observed for effects of dipstick proteinuria.

Our study derives from a representative sample of the UK community-dwelling older population, with systematic testing of serum creatinine and dipstick proteinuria at baseline and systematic follow-up for hospitalization during 2 years after these measurements. Our findings are not applicable to people living in nursing homes. Competing risks may have led to underestimation of the true effect of eGFR and dipstick proteinuria on hospitalization. A quarter of participants died during the follow-up period; 7% died without being hospitalized, and 18%, with at least 1 admission. Because both low eGFR and proteinuria are associated with higher risk of death, the risk of hospital admission or multiple admissions in those who died is different from those with low eGFR who did not die (competing risks). We used Cox regression rather than Poisson regression because of the limitations of Poisson. A Poisson approach would have assumed: (1) a constant rate of hospitalization during a given observation period (and thus ignored the issue of removal of those at highest risk of the hospitalization or at risk of death during follow-up), and (2) independence of risk of subsequent hospitalization from having had previous hospitalizations.

We were able to collect hospital admission data in only the first 2 years of follow-up and therefore our study does not provide evidence for longer term risks of hospital admission. Measurement errors may have led to underestimation of associations of eGFR or dipstick proteinuria There may be some interlaboratory variation in the creatinine measurement method,^17^ introducing random variation in serum creatinine and eGFR values. However, at high creatinine levels, there is less variation^17^ and estimation formulas perform better,^18^ which means that errors associated with risk estimates for eGFR <30 mL/min/1.73 m^2^ are likely to be minimal. Similarly, dipstick proteinuria readings are variable and less precise than urinary protein or albumin-creatinine ratios or 24-hour urine collections.^19,20^ Use of a midstream urine sample and exclusion of patients with culture-positive urine may have partially compensated for errors in dipstick proteinuria. Of 20,934 older people invited to join this study, 15,336 participated, of whom 12,371 had data for both dipstick protein and eGFR. Complete data for all potential confounders were available for only 10,799. Hence, there remains the possibility of selection bias in the fully adjusted analysis. However, analyses of the larger sets of data (12,371) gave virtually identical results. We cannot exclude the possibility of confounding caused by unmeasured factors, but this is a well-characterized data set that enabled us to adjust for an extensive range of confounding variables, including socioeconomic deprivation.

Our results show that both eGFR <30 mL/min/1.73 m^2^ and dipstick proteinuria are associated with increased risk of subsequent hospitalization. We found no association at higher eGFRs or for those with eGFR >75 mL/min/1.73 m^2^. There is considerable interest in minimizing hospital admissions for older people.^21^ Our results show that decreased kidney function, particularly in the presence of proteinuria, identifies older people at high risk of subsequent hospital admission.

## Figures and Tables

**Table 1:** Baseline Characteristics for 12,371 Participants With Both eGFR and Urine Data

	eGFR (mL/min/1.73 m^2^)					*P* for Trend	Proteinuria		*P*^a^
	<30	30-44	45-59	60-74	≥75+		Present	Absent	
No. of participants	396	2,029	666	3,787	1,493		922	11,449	
Age (y)	84.1 ± 5.2	83.1 ± 5.0	81.4 ± 4.5	80.1 ± 4.1	79.4 ± 3.7	<0.001	81.3 ± 4.7	81.1 ± 4.6	0.08
Women	288 (73)	1,452 (72)	3,046 (65)	2,036 (54)	713 (48)	<0.001	494 (54)	7,041 (62)	<0.001
Jarman scores						<0.001^a^			0.00
Low	113 (29)	596 (29)	1,328 (28)	1,254 (33)	604 (40)		317 (34)	3,578 (31)	
Middle	110 (28)	562 (28)	1,342 (29)	1,027 (27)	286 (19)		202 (22)	3,125 (27)	
High	173 (44)	871 (43)	1,996 (43)	1,506 (40)	603 (40)		403 (44)	4,746 (41)	
History of DM	42 (11)	190 (9)	314 (7)	273 (7)	138 (9)	0.5	108 (12)	849 (7)	<0.001
History of HTN	201 (51)	817 (41)	1,617 (35)	1,128 (30)	402 (27)	<0.001	393 (43)	3,772 (33)	<0.001
History of MI or stroke	142 (36)	480 (24)	779 (17)	583 (16)	169 (11)	<0.001	191 (21)	1,962 (17)	0.007
Smoking history						<0.001^a^			0.04
Nonsmoker	169 (43)	879 (43)	1,941 (42)	1,278 (34)	513 (34)		321 (35)	4,459 (39)	
Ex-smoker	190 (48)	938 (46)	2,227 (48)	2,043 (54)	755 (51)		491 (53)	5,662 (50)	
Current smoker	37 (9)	206 (10)	485 (10)	456 (12)	222 (15)		110 (12)	1,296 (11)	
Alcohol use						<0.001^a^			0.1
Never	83 (22)	416 (21)	718 (16)	431 (12)	180 (12)		150 (17)	1,678 (15)	
Ex	19 (5)	157 (8)	253 (6)	219 (6)	63 (4)		62 (7)	649 (6)	
Current	281 (73)	1,403 (71)	3,568 (79)	3,039 (82)	1,214 (83)		682 (76)	8,823 (79)	
Waist-to-hip ratio quintile^b^						0.006^a^			0.1
1	52 (14)	344 (18)	883 (20)	753 (21)	318 (22)		169 (19)	2,181 (20)	
2	68 (19)	344 (18)	923 (21)	706 (20)	291 (21)		152 (17)	2,180 (20)	
3	75 (21)	380 (20)	862 (20)	731 (20)	282 (20)		183 (21)	2,147 (20)	
4	86 (24)	414 (22)	852 (19)	724 (20)	266 (19)		193 (22)	2,149 (20)	
5	79 (22)	396 (21)	874 (20)	677 (19)	260 (18)		189 (21)	2,097 (20)	
BP ≥140/90 mm Hg	232 (60)	1,263 (63)	3,047 (66)	2,391 (64)	901 (61)	0.3	617 (67)	7,217 (64)	0.03
Antihypertensive use	204 (52)	859 (43)	1,596 (35)	1,087 (29)	390 (26)	<0.001	360 (40)	3,776 (34)	<0.001
Statin use	6 (2)	16 (1)	35 (1)	23 (1)	5 (0)	0.02	11 (1)	74 (1)	0.05
NSAID use	65 (17)	275 (14)	487 (11)	405 (11)	156 (11)	<0.001	109 (12)	1,279 (11)	0.6
ACEi/ARB use	77 (20)	296 (15)	437 (10)	219 (6)	94 (6)	<0.001	100 (11)	1,023 (9)	0.06
Aspirin use	104 (27)	462 (23)	892 (19)	673 (18)	218 (15)	<0.001	191 (21)	2,158 (19)	0.2
Albumin quintile^c^						<0.001^a^			0.2
1	89 (23)	305 (15)	578 (12)	522 (14)	255 (17)		126 (14)	1,623 (14)	
2	77 (20)	401 (20)	877 (19)	675 (18)	314 (21)		188 (21)	2,156 (19)	
3	55 (14)	336 (17)	776 (17)	665 (18)	277 (19)		150 (16)	1,959 (17)	
4	70 (18)	467 (23)	1,065 (23)	834 (22)	268 (18)		180 (20)	2,524 (22)	
5	99 (25)	496 (25)	1,338 (29)	1,052 (28)	365 (25)		272 (30)	3,078 (27)	
Hemoglobin quintile^b^						<0.001^a^			0.00
1	182 (47)	523 (26)	798 (18)	534 (14)	227 (15)		213 (23)	2,051 (18)	
2	72 (19)	440 (22)	805 (18)	681 (18)	254 (17)		175 (19)	2,077 (19)	
3	46 (12)	342 (17)	881 (19)	756 (20)	315 (22)		163 (18)	2,177 (19)	
4	51 (13)	362 (18)	1,041 (23)	892 (24)	370 (25)		181 (20)	2,535 (23)	
5	33 (9)	322 (16)	1,031 (23)	844 (23)	299 (20)		175 (19)	2,354 (21)	
Phosphate quintile^b^						<0.001^a^			0.4
1	41 (11)	336 (18)	865 (20)	676 (20)	266 (19)		153 (18)	2,031 (19)	
2	49 (13)	331 (18)	849 (20)	747 (22)	287 (21)		185 (22)	2,078 (20)	
3	57 (15)	340 (18)	828 (19)	708 (20)	270 (20)		154 (18)	2,049 (20)	
4	81 (22)	377 (20)	877 (21)	710 (21)	286 (21)		165 (20)	2,166 (21)	
5	146 (39)	479 (26)	844 (20)	616 (18)	268 (19)		186 (22)	2,167 (21)	
Partially/fully dependent	225 (57)	847 (42)	1,311 (28)	834 (22)	309 (21)	<0.001	292 (32)	3,234 (28)	0.03

Cognitive impairment	98 (26)	453 (23)	770 (17)	531 (14)	214 (15)	<0.001	180 (20)	1,886 (17)	0.02
Depressed	37 (9)	187 (9)	331 (7)	235 (6)	113 (8)	0.002	94 (10)	809 (7)	<0.001
Health perception = poor	16 (4)	40 (2)	69 (1)	62 (2)	35 (2)	0.5	23 (3)	199 (2)	0.1
Physically active						<0.001^a^			0.04
Very	41 (10)	406 (20)	1,284 (28)	1,186 (32)	465 (31)		232 (25)	3,150 (28)	
Fairly	183 (47)	1,014 (50)	2,418 (52)	1,914 (51)	736 (50)		454 (50)	5,811 (51)	
Not very	128 (33)	474 (24)	786 (17)	547 (15)	213 (14)		191 (21)	1,957 (17)	
Not at all	41 (10)	118 (6)	156 (3)	108 (3)	70 (5)		38 (4)	455 (4)	
≥2 falls at home in last 6 mo	55 (14)	205 (10)	365 (8)	223 (6)	125 (8)	<0.001	85 (9)	888 (8)	0.1

*Note:* Values shown as mean ± standard deviation or number (percentage); unless otherwise indicated, column percentages are shown. Proteinuria presence assessed using dipstick positivity. Abbreviations: ACEi, angiotensin-converting enzyme inhibitor; ARB, angiotensin receptor blocker; BP, blood pressure; DM, diabetes mellitus; eGFR, estimated glomerular filtration rate; HTN, hypertension; MI, myocardial infarction; NSAID, nonsteroidal anti-inflammatory drug.

a χ^2^ test for association.

b Sex-specific.

c Sex- and assay-specific.

**Table 2:** Rates of Subsequent Hospital Admission According to Selected Participants' Baseline Characteristics

	No. Hospitalized	Hospitalization Rate per 100 Person-Years (95% CI)	*P*^a^
Age (y)			<0.001
75-80	1,376	11.8 (12.1-13.5)	
80-85	1,082	16.0 (15.0-16.9)	
85-90	608	19.0 (17.5-20.6)	
≥90	193	19.1 (16.6-22.0)	
Sex			<0.001
Male	1,390	16.8 (15.9-17.7)	
Female	1,869	13.9 (13.3-14.5)	
eGFR (mL/min/1.73 m^2^)			<0.001
≥75	390	14.8 (13.4-16.3)	
60-74	923	13.6 (12.8-14.5)	
45-59	1,182	14.3 (13.5-15.2)	
30-44	617	17.7 (16.4-19.2)	
<30	417	23.7 (20.2-27.9)	
Proteinuria^b^			<0.001
Negative	2,748	14.4 (13.9-15.0)	
Positive	290	20.1 (17.9-22.5)	
Missing	221	17.9 (15.7-20.4)	
Jarman scores			<0.001
Low	893	12.8 (12.0-13.7)	
Middle	871	15.1 (14.1-16.1)	
High	1,495	16.6 (15.7-17.4)	
History of DM			<0.001
No	2,945	14.6 (14.1-15.1)	
Yes	314	19.6 (17.6-21.9)	
History of HTN			0.3
No	2,107	14.8 (14.2-15.4)	
Yes	1,121	15.4 (14.5-16.3)	
History of MI or stroke			<0.001
No	2,473	13.7 (13.1-14.2)	
Yes	758	21.8 (20.3-23.4)	
Smoking history			<0.001
Nonsmoker	1,190	13.9 (13.2-14.8)	
Ex-smoker	1,621	15.1 (14.3-15.8)	
Current smoker	436	18.2 (16.6-20.0)	
Alcohol use			0.2
Never	490	15.1 (13.9-16.5)	
Ex	224	18.5 (16.3-21.1)	
Current	2,461	14.7 (14.1-15.3)	
Waist-to-hip ratio quintile^c^			<0.001
1	550	13.3 (12.2-14.4)	
2	585	14.0 (12.9-15.2)	
3	581	14.0 (12.9-15.2)	
4	642	15.8 (14.7-17.1)	
5	651	16.2 (15.0-17.5)	
			
BP (mm Hg)			0.01
<140/90	1,232	16.0 (15.1-16.9)	
≥140/90	2,005	14.5 (13.8-15.1)	
Antihypertensive use			0.002
No	2,060	14.5 (13.8-15.1)	
Yes	1,160	16.2 (15.3-17.2)	
Statin use			0.6
No	3,200	15.1 (14.5-15.6)	
Yes	20	13.5 (8.7-21.0)	
NSAID use			<0.001
No	2,800	14.7 (14.2-15.3)	
Yes	420	17.6 (16.0-19.4)	
ACEi/ARB use			<0.001
No	2,835	14.5 (14.0-15.0)	
Yes	385	21.2 (19.2-23.4)	
Hemoglobin quintile^c^			<0.001
1	784	21.5 (20.0-23.0)	
2	639	16.3 (15.1-17.6)	
3	578	13.7 (12.7-14.9)	
4	619	12.7 (11.7-13.7)	
5	566	12.3 (11.3-13.4)	
Phosphate quintile^c^			0.01
1	544	13.9 (12.8-15.1)	
2	608	15.5 (14.3-16.8)	
3	597	15.6 (14.4-16.9)	
4	612	14.9 (13.7-16.1)	
5	686	16.9 (15.7-18.3)	
Albumin quintile^d^			<0.001
1	554	19.4 (17.9-21.1)	
2	624	15.1 (13.9-16.4)	
3	559	15.1 (13.9-16.4)	
4	695	14.4 (13.3-15.5)	
5	793	13.1 (12.2-14.1)	
Partially/fully dependent			<0.001
ADL <2	1,972	12.3 (11.8-12.9)	
ADL ≥2	1,281	22.4 (21.2-23.7)	
Cognitive impairment			<0.001
MMSE ≤23	660	18.7 (17.3-20.2)	
MMSE >23	2,512	14.1 (13.6-14.7)	
Depressed			<0.001
GDS ≤5	2,882	14.4 (13.9-14.9)	
GDS >5	327	22.2 (19.9-24.7)	
Health perception			<0.001
Not poor	3,121	14.7 (14.1-15.2)	
Poor	109	36.3 (30.0-43.7)	
			
Physically active			<0.001
Very	649	10.4 (9.6-11.2)	
Fairly	1,568	14.0 (13.4-14.8)	
Not very	800	23.2 (21.6-24.8)	
Not at all	212	29.6 (25.8-33.8)	
Falls at home in last 6 mo			<0.001
<2	2,878	14.3 (13.8-14.9)	
≥2	366	23.3 (21.0-25.8)	
Missing CV data			0.001
Yes	580	16.9 (15.6-18.4)	
No	2,679	14.6 (14.1-15.2)	

Abbreviations: ACEi, angiotensin-converting enzyme inhibitor; ADL, activities of daily living; ARB, angiotensin receptor blocker; BP, blood pressure; CI, confidence interval; CV, cardiovascular; DM, diabetes mellitus; eGFR, estimated glomerular filtration rate; GDS, Geriatric Depression Scale; HTN, hypertension; MI, myocardial infarction; MMSE, Mini-Mental State Examination; NSAID, nonsteroidal anti-inflammatory drug.

a Trend in rate ratio.

b Midstream urine positive.

c Sex-specific.

d Sex- and assay-specific. For albumin, 39 of 45 laboratories used bromocresol green, the rest used bromocresol purple, and quintiles were derived by assay and sex.

**Table 3:** Age-Adjusted Ratios of Hospital Admission Rates Across Categories of eGFR in Men and Women Stratified by Presence of Proteinuria

Proteinuria	eGFR (mL/min/1.73 m^2^)	Men	Women
Absent	≥75	1.11 (0.94-1.31)	1.12 (0.92-1.35)
	60-74	1.00 (reference)	1.00 (reference)
	45-59	0.99 (0.86-1.13)	1.08 (0.95-1.23)
	30-44	1.21 (0.99-1.47)	1.19 (1.03-1.39)
	<30	1.18 (0.76-1.85)	1.52 (1.18-1.95)
Present	≥75	0.73 (0.42-1.28)	1.09 (0.46-2.59)
	60-74	1.00 (reference)	1.00 (reference)
	45-59	0.64 (0.41-1.01)	1.23 (0.77-1.95)
	30-44	0.92 (0.58-1.46)	2.19 (1.35-3.54)
	<30	1.21 (0.65-2.25)	2.91 (1.57-5.40)

*Note:* Values shown as rate ratio (95% confidence interval). Mantel-Haenszel tests for interaction between sex and eGFR and between eGFR and proteinuria were nonsignificant. Proteinuria presence was assessed using dipstick. Abbreviation: eGFR, estimated glomerular filtration rate.

**Table 4:** Effects of Sequential Adjustments in Complete-Case Subset of Data on Associations of eGFR and Proteinuria With Subsequent Hospitalization

Model: Category	<6 mo (n = 10,977)	6-24 mo (n = 10,042)
**Effect of eGFR Alone (not adjusted for proteinuria)**		
1: age, sex adjusted		
eGFR ≥75	1.11 (0.91-1.36)	1.04 (0.88-1.23)
eGFR = 60-74	1.00 (reference)	1.00 (reference)
eGFR = 45-59	1.11 (0.93-1.34)	1.01 (0.92-1.11)
eGFR = 30-44	1.36 (1.11-1.65)	1.19 (1.01-1.41)
eGFR <30	2.17 (1.59-2.96)	1.53 (1.18-1.99)
2: as above and adjusted for CVD risk factors^a^		
eGFR ≥75	1.11 (0.91-1.35)	1.08 (0.91-1.27)
eGFR = 60-74	1.00 (reference)	1.00 (reference)
eGFR = 45-59	1.08 (0.90-1.30)	0.97 (0.88-1.07)
eGFR = 30-44	1.17 (0.95-1.43)	1.04 (0.89-1.21)
eGFR <30	1.66 (1.21-2.27)	1.23 (0.95-1.59)
**eGFR and Proteinuria Adjusted for Each Other**		
1: age, sex adjusted		
eGFR^b^ ≥75	1.11 (0.91-1.36)	1.04 (0.88-1.23)
eGFR^b^ = 60-74	1.00 (reference)	1.00 (reference)
eGFR^b^ = 45-59	1.11 (0.92-1.33)	1.00 (0.91-1.10)
eGFR^b^ = 30-44	1.34 (1.10-1.64)	1.18 (1.00-1.40)
eGFR^b^ <30	2.10 (1.54-2.85)	1.48 (1.14-1.93)
Proteinuria^c^	1.30 (1.02-1.66)	1.31 (1.07-1.60)
2: as above, and adjusted for CVD risk factors^a^		
eGFR^b^ ≥75	1.11 (0.91-1.35)	1.08 (0.91-1.28)
eGFR^b^ = 60-74	1.00 (reference)	1.00 (reference)
eGFR^b^ = 45-59	1.08 (0.90-1.30)	0.97 (0.88-1.07)
eGFR^b^ = 30-44	1.16 (0.95-1.42)	1.03 (0.88-1.20)
eGFR^b^ <30	1.62 (1.18-2.21)	1.20 (0.93-1.54)
Proteinuria^c^	1.26 (0.99-1.59)	1.28 (1.05-1.55)

*Note:* Values shown as hazard ratio (95% confidence interval). eGFRs are given in mL/min/1.73 m^2^. Abbreviations: CVD, cardiovascular disease; eGFR, estimated glomerular filtration rate.

a In addition to age and sex, model 2 was adjusted for Jarman deprivation score (low, middle, and high), smoking status (current, ex-, or never smoker), alcohol (never, ex-, and current drinker), self-reported physical activity (very active, fairly, not very, and not at all), waist-to-hip ratio (sex-specific quintiles), and comorbid conditions using self-reported history of CVD (heart attack or stroke) and diabetes. Hypertension was modeled separately as physician-diagnosed hypertension, average blood pressure >140/90 mm Hg, or use of antihypertensive drugs. Cardiovascular drugs were statins or aspirin. We adjusted for angiotensin-converting enzyme inhibitors and angiotensin II receptor blockers separately from other antihypertensive agents.

b eGFR adjusted for proteinuria.

c Dipstick-positive proteinuria adjusted for eGFR.

**Table 5:** Effects of Sequential Adjustments in Complete-Case Subset of Data on Association of eGFR and Proteinuria (modeled separately) With Hospital Admissions During 2-Year Follow-up

Model: Category	1 Admission vs None	≥2 Admissions vs None
**Effect of eGFR Alone**		
1: age, sex adjusted		
eGFR ≥75	1.11 (0.93-1.34)	0.92 (0.67-1.27)
eGFR = 60-74	1.00 (reference)	1.00 (reference)
eGFR = 45-59	1.03 (0.91-1.17)	1.03 (0.85-1.26)
eGFR = 30-44	1.21 (1.02-1.44)	1.31 (1.03-1.68)
eGFR <30	1.37 (1.02-1.84)	2.38 (1.63-3.48)
2: as above and adjusted for CVD risk factors^a^		
eGFR ≥75	1.14 (0.94-1.38)	0.96 (0.70-1.33)
eGFR = 60-74	1.00 (reference)	1.00 (reference)
eGFR = 45-59	1.00 (0.88-1.13)	0.96 (0.78-1.19)
eGFR = 30-44	1.07 (0.91-1.27)	1.04 (0.80-1.35)
eGFR <30	1.10 (0.81-1.49)	1.60 (1.08-2.36)
**Effect of Proteinuria Alone**		
1: age, sex adjusted		
Proteinuria absent	1.00 (reference)	1.00 (reference)
Proteinuria present	1.35 (1.09-1.68)	1.51 (1.17-1.95)
2: as above and adjusted for CVD risk factors^a^		
Proteinuria absent	1.00 (reference)	1.00 (reference)
Proteinuria present	1.31 (1.06-1.62)	1.41 (1.12-1.76)
**eGFR and Proteinuria Adjusted for Each Other**		
1: age, sex adjusted		
eGFR^b^ ≥75	1.12 (0.93-1.34)	0.92 (0.67-1.27)
eGFR^b^ = 60-74	1.00 (reference)	1.00 (reference)
eGFR^b^ = 45-59	1.03 (0.91-1.16)	1.03 (0.84-1.25)
eGFR^b^ = 30-44	1.20 (1.01-1.42)	1.29 (1.01-1.65)
eGFR^b^ <30	1.32 (0.98-1.78)	2.27 (1.55-3.32)
Proteinuria present^c^	1.33 (1.06-1.66)	1.42 (1.11-1.82)
2: as above and adjusted for CVD risk factors^a^		
eGFR^b^ ≥75	1.14 (0.94-1.38)	0.97 (0.70-1.33)
eGFR^b^ = 60-74	1.00 (reference)	1.00 (reference)
eGFR^b^ = 45-59	1.00 (0.88-1.13)	0.96 (0.78-1.18)
eGFR^b^ = 30-44	1.06 (0.90-1.26)	1.03 (0.79-1.33)
eGFR^b^ <30	1.06 (0.78-1.44)	1.54 (1.04-2.27)
Proteinuria present^c^	1.31 (1.06-1.62)	1.37 (1.10-1.71)

*Note:* N = 10,977. Values shown as odds ratio (95% confidence interval). eGFRs are given in mL/min/1.73 m^2^. Proteinuria assessed as dipstick positivity. Abbreviations: CVD, cardiovascular disease; eGFR, estimated glomerular filtration rate.

a In addition to age and sex, model 2 was adjusted for Jarman deprivation score (low, middle, and high), smoking status (current, ex, or never smoker), alcohol (never, ex-, and current drinker), self-reported physical activity (very active, fairly, not very, and not at all), waist-to-hip ratio (sex-specific quintiles), and comorbid conditions using self-reported history of CVD (heart attack or stroke) and diabetes. Hypertension was modeled separately as physician-diagnosed hypertension, average blood pressure >140/90 mm Hg, or use of antihypertensive drugs. Cardiovascular drugs were statins or aspirin. We adjusted for angiotensin-converting enzyme inhibitors and angiotensin II receptor blockers separately from other antihypertensive agents.

b eGFR adjusted for proteinuria.

c Adjusted for eGFR.

## References

[1] Roderick P.J., Atkins R.J., Smeeth L. (2009). CKD and mortality risk in older people: a community-based population study in the United Kingdom. Am J Kidney Dis.

[2] Go A.S., Chertow G.M., Fan D. (2004). Chronic kidney disease and the risks of death, cardiovascular events, and hospitalization. N Engl J Med.

[3] Levey A.S., Eckardt K.U., Tsukamoto Y. (2005). Definition and classification of chronic kidney disease: a position statement from Kidney Disease: Improving Global Outcomes (KDIGO). Kidney Int.

[4] Roderick P.J., Atkins R.J., Smeeth L. (2008). Detecting chronic kidney disease in older people; what are the implications?. Age Ageing.

[5] Fletcher A.E., Jones D.A., Bulpitt C.J., Tulloch A.J. (2002). The MRC Trial of Assessment and Management of Older People in the Community: objectives, design and interventions [ISRCTN23494848]. BMC Health Serv Res.

[6] Fletcher A.E., Price G.M., Ng E.S. (2004). Population-based multidimensional assessment of older people in UK general practice: a cluster-randomised factorial trial. Lancet.

[7] Jarman B. (1984). Underprivileged areas: validation and distribution of scores. BMJ.

[8] Folstein M., Folstein S., McHugh P. (1975). “Mini-Mental State”: A practical method for grading the cognitive state of patients for the clinician. J Psychiatr Res.

[9] Sheik J., Yesavage J. (1986). Geriatric Depression Scale (GDS): recent evidence and development of a shorter version. Clin Gerontol.

[10] Levey A.S., Stevens L.A., Schmid C.H. (2009). A new equation to estimate glomerular filtration rate. Ann Intern Med.

[11] Levey A.S., Stevens L.A. (2010). Estimating GFR using the CKD Epidemiology Collaboration (CKD-EPI) creatinine equation: more accurate GFR estimates, lower CKD prevalence estimates, and better risk predictions. Am J Kidney Dis.

[12] Coresh J. (2009). CKD prognosis: beyond the traditional outcomes. Am J Kidney Dis.

[13] Levey A.S., Coresh J., Greene T. (2007). Expressing the Modification of Diet in Renal Disease Study equation for estimating glomerular filtration rate with standardized serum creatinine values. Clin Chem.

[14] James M.T., Quan H., Tonelli M. (2009). CKD and risk of hospitalization and death with pneumonia. Am J Kidney Dis.

[15] Laliberte F., Bookhart B.K., Vekeman F. (2009). Direct all-cause health care costs associated with chronic kidney disease in patients with diabetes and hypertension: a managed care perspective. J Manag Care Pharm.

[16] Winkelmayer W.C., Schneeweiss S., Mogun H. (2005). Identification of individuals with CKD from Medicare claims data: a validation study. Am J Kidney Dis.

[17] Miller W.G., Myers G.L., Ashwood E.R. (2005). Creatinine measurement: state of the art in accuracy and interlaboratory harmonization. Arch Pathol Lab Med.

[18] Murthy K., Stevens L.A., Stark P.C., Levey A.S. (2005). Variation in the serum creatinine assay calibration: a practical application to glomerular filtration rate estimation. Kidney Int.

[19] Hinberg I.H., Katz L., Waddell L. (1978). Sensitivity of in vitro diagnostic dipstick tests to urinary protein. Clin Biochem.

[20] Lambers Heerspink H.J., Brantsma A.H., de Zeeuw D. (2008). Albuminuria assessed from first-morning-void urine samples versus 24-hour urine collections as a predictor of cardiovascular morbidity and mortality. Am J Epidemiol.

[21] Murphy E. (2004). Case management and community matrons for long term conditions. BMJ.

